# Precision Medicine for Frontotemporal Dementia

**DOI:** 10.3389/fpsyt.2019.00075

**Published:** 2019-02-21

**Authors:** Mu-N Liu, Chi-Ieong Lau, Ching-Po Lin

**Affiliations:** ^1^Institute of Brain Science, National Yang-Ming University, Taipei, Taiwan; ^2^Department of Psychiatry, Taipei Veterans General Hospital, Taipei, Taiwan; ^3^Department of Neurology, Memory and Aging Centre, University of California, San Francisco, San Francisco, CA, United States; ^4^Department of Neurology, Shin Kong Wu Ho-Su Memorial Hospital, Taipei, Taiwan; ^5^Applied Cognitive Neuroscience Group, Institute of Cognitive Neuroscience, University College London, London, United Kingdom; ^6^College of Medicine, Fu-Jen Catholic University, Taipei, Taiwan; ^7^Institute of Neuroscience, National Yang-Ming University, Taipei, Taiwan; ^8^Aging and Health Research Center, National Yang Ming University, Taipei, Taiwan

**Keywords:** frontotemporal dementia, frontotemporal lobar degeneration, genetics, precision medicine, neuroimaging, primary progressive aphasia

## Abstract

Frontotemporal dementia (FTD) is a common young-onset dementia presenting with heterogeneous and distinct syndromes. It is characterized by progressive deficits in behavior, language, and executive function. The disease may exhibit similar characteristics to many psychiatric disorders owing to its prominent behavioral features. The concept of precision medicine has recently emerged, and it involves neurodegenerative disease treatment that is personalized to match an individual's specific pattern of neuroimaging, neuropathology, and genetic variability. In this paper, the pathophysiology underlying FTD, which is characterized by the selective degeneration of the frontal and temporal cortices, is reviewed. We also discuss recent advancements in FTD research from the perspectives of clinical, imaging, molecular characterizations, and treatment. This review focuses on the approach of precision medicine to manage the clinical and biological complexities of FTD.

## Introduction

Frontotemporal dementia (FTD) is an insidious neurodegenerative clinical syndrome that is characterized by progressive disturbances in behavior as well as deficits in executive function and language. FTD is a common early-onset dementia (occurring in patients aged < 65 years), has a prevalence rate of 3–26%, and is one of the most common forms of dementia across all age groups (1). Arnold Pick, a Czech psychiatrist, first identified the clinical syndrome of FTD in 1892 (2). He described a patient with aphasia, focal frontal and temporal lobar atrophy, and presenile dementia. Alois Alzheimer, a German psychiatrist and neuropathologist, later characterized Pick bodies as being associated with FTD and named the disorder Pick's disease in 1911 (3). Although, the term Pick's disease initially referred to both the clinical syndrome and the pathological diagnosis, modern nomenclature designates Pick's disease as only the pathological diagnosis, whereas a clinical diagnosis for prominent behavioral changes is known as behavioral-variant FTD (bvFTD). Mesulam described primary progressive aphasia (PPA), the language subtype of FTD, in 1982 (4). Revised diagnostic criteria were issued in 2011 (5, 6).

Precision medicine, also called “personalized medicine” or “individualized medicine,” is a rapidly advancing field in medical, clinical, and research settings. It aims to optimize the effectiveness of disease prevention and treatment and simultaneously minimize side effects in individuals who are less likely to respond to a particular therapy, by considering an individual's specific makeup with regard to genetics, biomarkers, phenotype, and psychosocial characteristics. In this review, we discuss the precision medicine of FTD, from clinical phenotypes, epidemiology, genetics, neuroimaging to neuropathological biomarkers. We further review recent advancements in therapeutic strategies and potential personalized treatment for FTD (7–13). This review improves the understanding of accurate diagnosis and personalized effective disease treatment strategies.

## Cognitive and Behavioral Markers

FTD is an umbrella term for three recognizable clinical syndromes, namely bvFTD, semantic-variant PPA (svPPA), and non-fluent-variant PPA (nfvPPA) (Table 1). FTD also frequently overlaps clinically with three neurodegenerative diseases that exhibit motor deficits, namely corticobasal degeneration (CBD), progressive supranuclear palsy (PSP), and amyotrophic lateral sclerosis (14).

**Table 1:** Clinical features of bvFTD, svPPA, and nfvPPA (5, 6).

**Syndrome**	**Possible diagnosis with clinical evidence**	**Probable diagnosis with imaging evidence**	**Definite diagnosis with pathological or genetic support**	**Exclusionary criteria**
bvFTD	At least three of the following: Behavioral disinhibition (socially inappropriate behavior; Loss of manners or decorum; impulsive, rash or careless actions) within the first 3 years. Apathy or inertia within the first 3 years Lack of empathy or sympathy (loss of response to other people's needs and feelings; loss of social interest, interrelatedness or personal warmth) within the first 3 years Perseverations, stereotypies or compulsions (simple repetitive movements; complex, compulsive or ritualistic behaviors; stereotypy of speech) within the first 3 years Dietary habit changes or hyperorality (altered food preferences; binge eating, increased consumption of alcohol or cigarettes; oral exploration or consumption of inedible objects) Executive-predominant deficits on neuropsychological profile with relative sparing of episodic memory and visuospatial skills	All of the following: Meets possible diagnostic criteria Significant functional deficit (according to Clinical Dementia Rating Scale, Functional Activities Questionnaire scores, or caregiver report) Imaging consistent with bvFTD (predominant frontal and/or anterotemporal atrophy, hypoperfusion and/or hypometabolism)	All of the following: Meets possible or probable diagnostic criteria Histopathological proof of FTLD pathology and/or existence of a known pathogenic mutation	Deficits or disturbances are not better explained by other disorders (neurodegenerative, non-degenerative nervous system, psychiatric, or medical diseases)
svPPA	Both of the following core features: Impaired confrontation naming Impaired single-word comprehension At least three of the following four features: Impaired object knowledge (particularly for low familiar or frequent items) Surface dyslexia or dysgraphia Spared repetition Spared grammar and motor speech (speech production)	All of the following: Meets possible diagnostic criteria Imaging consistent with svPPA (predominant anterior temporal lobe atrophy, hypoperfusion and/or hypometabolism)	All of the following: Meets possible or probable diagnostic criteria Histopathological proof of FTLD pathology and/or existence of a known pathogenic mutation	Deficits are not better explained by other disorders (non-degenerative nervous system, psychiatric, or medical diseases) Prominent initial symptoms are not episodic memory, visuospatial impairments, or behavioral disturbance
nfvPPA	At least one of the following two core features: Agrammatism Effortful, halting speech with inconsistent sound errors and distortions (apraxia of speech) At least two of the following three features: Impaired comprehension of syntactically complex sentences Spared single-word comprehension Spared object knowledge	All of the following: Meets possible diagnostic criteria Imaging consistent with nfvPPA (predominant left posterior frontoinsular atrophy, hypoperfusion and/or hypometabolism)	All of the following: Meets possible or probable diagnostic criteria Histopathological proof of FTLD pathology and/or existence of a known pathogenic mutation	Deficits are not better explained by other disorders (non-degenerative nervous system, psychiatric, or medical diseases) Prominent initial symptoms are not episodic memory, visuospatial impairments, or behavioral disturbance

*BvFTD, behavioral-variant frontotemporal dementia; svPPA, semantic-variant primary progressive aphasia; and nfvPPA, non-fluent variant primary progressive aphasia*.

### Behavioral-Variant Frontotemporal Dementia

The symptoms of bvFTD include progressive personality and behavioral changes, apathy, and disinhibition in interpersonal interactions. Patients may experience early changes in disinhibition, stereotypic behavior, alterations in food preferences and eating behavior, alterations in empathy, apathy, and dysexecutive symptoms (5, 15). Some of these early symptoms, such as decreased empathy, may have diagnostic value for bvFTD, but they have not been ascertained in clinical practice. Apathy may manifest as reduced interest in work, hobbies, social interaction, and hygiene; however, apathy can be misdiagnosed as depression.

Symptoms similar to those detected in psychiatric disorders are frequently observed in patients with bvFTD. Thus, discriminating the behavioral features of bvFTD from those of primary psychiatric disorders such as depression, schizophrenia, bipolar disorder, and borderline personality disorder may be challenging (16, 17). Although psychotic symptoms such as hallucinations and delusions are rare in bvFTD, cases of these symptoms have been reported (17), particularly in patients carrying the chromosome 9 open reading frame 72 *(C9orf72)* repeat expansion (18).

### Primary Progressive Aphasia

Patients with PPA exhibit a progressive decline in linguistic skills during the early phase of the disease. Language dysfunction is the main symptom during the first 2 years of PPA. Deficits in object naming, syntax, or word comprehension may become apparent during conversation or may be identified using speech and language assessment. The subtypes of PPA are differentiated by specific types of speech or language deficits. The three PPA subtypes are the semantic, non-fluent, and logopenic variants (19). Each subtype has a distinct pattern of language deficits. Naming difficulty is common to all three subtypes; therefore, it is not a distinguishing feature. The non-fluent (or agrammatic) variant and the semantic variant are classified as FTD, whereas the logopenic variant, most often associated with temporoparietal atrophy, is typically due to underlying Alzheimer's pathology; hence, it is not discussed in this review.

### Semantic-Variant Primary Progressive Aphasia

In svPPA, a syndrome characterized by semantic aphasia and associative agnosia, anterior temporal lobe degeneration disrupts semantic memory (Table 1) (6). Anomia and single-word comprehension deficits, starting with low-frequency items, are essential for diagnosis (20). In contrast to patients with nfvPPA, those with svPPA maintain fluent speech and correct grammar during the early stages of this disease. Early symptoms of semantic PPA include anomia, word-finding difficulties, and repetitive speech, whereas early behavioral syndrome presents with irritability and emotional distance or coldness.

### Non-fluent/Agrammatic-Variant Primary Progressive Aphasia

Articulation deficits resulting in slow, labored, and halting speech production as well as incorrect grammar or syntax (agrammatism) characterize nfvPPA. The core criteria of nfvPPA are agrammatism and effortful speech, and at least one of the criteria should be present (Table 1) (6). Patients tend to exhibit motor speech disorders characterized by a slow speech rate, abnormal prosody, and distorted sound substitutions, additions, repetitions, and prolongations, which are occasionally accompanied by groping, trial-and-error articulatory movements (21), or agrammatic errors. Repetition is less impaired than is spontaneous speech, and semantic knowledge for words typically remains well-preserved throughout the disease process.

### Motor Symptoms

The three FTD-spectrum motor syndromes are FTD with motor neuron disease (FTD-MND) and two variants with parkinsonism, namely corticobasal syndrome (CBS) and progressive supranuclear palsy syndrome (PSP-S). Up to 15% of patients with FTD have concomitant MND, and nearly 30% of patients present with mild features of MND (9, 22). MND may include upper motor neuron signs (hyperreflexia, extensor plantar response, and spasticity), lower motor neuron signs (weakness, muscle atrophy, and fasciculations), dysarthria, dysphagia, and pseudobulbar affect (22). Up to 20% of patients with FTD present with parkinsonism, which is most often observed in patients with bvFTD, followed by those with nfvPPA (23). Patients with FTD may exhibit features of CBS or PSP-S. CBS is a heterogeneous syndrome featuring behavioral, cognitive, and motor changes. The clinical criteria for probable CBS include asymmetric presentation with any two symptoms among (A) limb rigidity or akinesia, (B) limb dystonia, and (C) limb myoclonus, as well as any two symptoms among (D) orobuccal or limb apraxia, (E) cortical sensory deficit, and (F) alien limb phenomena (more than simple levitation) (24). Finally, PSP-S is characterized by atypical parkinsonism with axial and symmetrical rigidity, supranuclear gaze palsy (most prominent in the vertical plane), decreased saccadic velocity, early postural instability with falls, and prominent frontal lobe dysfunction (25, 26).

Taken together, the vast heterogeneity and overlap of clinical phenotypes in FTD often poses diagnostic challenges for clinicians, in particular the presenting psychiatric symptoms that may easily be mistaken for psychiatric disorders. The accurate diagnosis of each subtype of FTD, therefore, requires a precision medicine approach.

## Imaging Biomarkers

**Figure 1:**
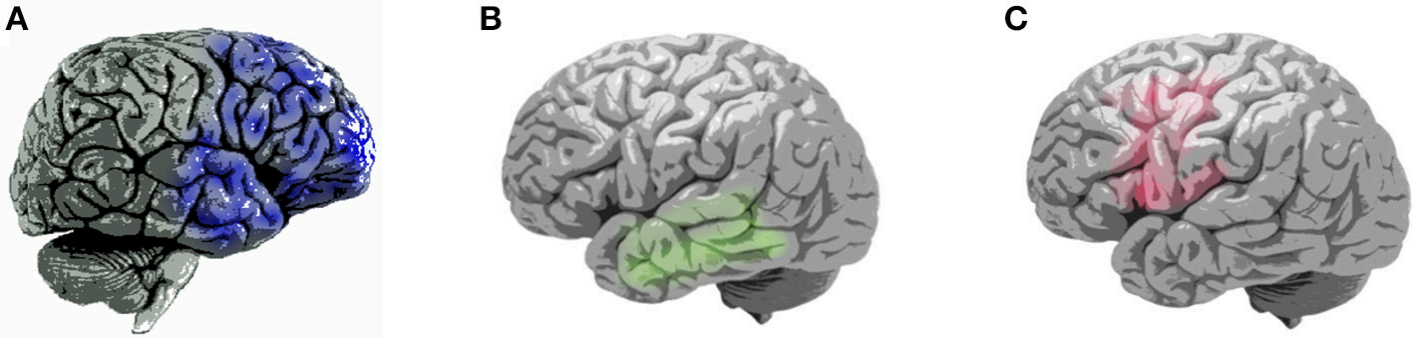
Patterns of brain atrophy in clinical subtypes of frontotemporal dementia (7–9). **(A)** Areas of brain atrophy in behavioral-variant frontotemporal dementia (blue), right hemisphere lateral view; **(B)** Areas of brain atrophy in semantic-variant primary progressive aphasia(green), left hemisphere lateral view; **(C)** Areas of brain atrophy in non-fluent variant primary progressive aphasia, left hemisphere lateral view.

Neuroimaging has the potential to aid the differential diagnosis of FTD. For example, FTD is characterized by predominant frontal or temporal atrophy, particularly in the frontoinsular region, as revealed by structural brain imaging (Figure 1; Table 2) (8). Using voxel-based morphometry, Rosen et al. demonstrated that core neuropsychiatric symptoms of bvFTD, including apathy, disinhibition, and aberrant motor behavior, are localized to the right frontal structures. Moreover, atrophy in the right-hemispheric anterior cingulate cortex and adjacent ventromedial superior frontal gyrus, posterior ventromedial prefrontal cortex, lateral middle frontal gyrus, caudate head, orbitofrontal cortex, and anterior insula was correlated with symptom severity (27). Very mild bvFTD targets paralimbic networks, including the anterior cingulate, insular, medial frontal, and orbitofrontal cortices (28). Specifically, atrophy of the right ventromedial superior frontal gyrus was associated with apathy; atrophy of the right ventromedial prefrontal cortex was associated with disinhibition (27); and atrophy of the dorsolateral prefrontal was associated with executive deficit (7). A widespread alteration in white matter connectivity between the frontal and temporal lobes was noted using diffusion tensor imaging. The uncinate fasciculus, anterior parts of the superior and inferior longitudinal fasciculi, genu of the corpus callosum, cingulum, and inferior fronto-occipital fasciculus were affected (Table 2) (10, 29).

**Table 2:** Imaging and pathological characteristics of frontotemporal dementia (7–9).

**Three types FTD**		**Genetic and pathological characteristics**	**Imaging characteristics**
Behavior variant	bvFTD	*C9orf72, MAPT*, and *GRN* mutations; tau and TDP-43 proteinopathy	Prefrontal and anterior temporal cortex loss, particularly in the right hemisphere; reduced white matter fractional anisotropy in uncinate fasciculus; striatum, thalamus, anterior cingulate, and insula atrophy; reduced fractional anisotropy in superior and inferior longitudinal fasciculi, inferior fronto-occipital fasciculus, genu of corpus callosum and cingulum
Language variant	svPPA	Rare genetic mutations; tau and TDP-43 (majority) proteinopathy	Brain atrophy in inferior temporal and fusiform gyri, temporal pole, parahippocampal cortex, entorhinal cortex, particularly in the left hemisphere; reduced fractional anisotropy in left superior longitudinal fasciculus and corpus callosum; brain atrophy also affecting anterior cingulate, orbitofrontal, inferior frontal, and insular cortices; reduced fractional anisotropy in left cingulum, left orbitofrontal, inferior frontal, anterior temporal, inferior parietal white matter regions
	nfvPPA	*GRN* mutations; tau (majority), and TDP-43 proteinopathy	Brain atrophy in the inferior frontal, prefrontal and temporal cortices, caudate and putamen, particularly in the left hemisphere*;* reduced fractional anisotropy in left uncinate fasciculus, corpus callosum, cingulum, and inferior longitudinal fasciculus tract

*BvFTD, behavioral-variant frontotemporal dementia; svPPA, semantic-variant primary progressive aphasia; nfvPPA, non-fluent variant primary progressive aphasia; C9orf72, chromosome 9 open reading frame 72; MAPT, microtubule-associated protein tau; GRN, progranulin mutations; and TDP-43, transactive response (TAR) DNA-binding protein of 43 kDa*.

By contrast, language symptoms are usually localized to the left hemisphere and are associated with a deficit of the language circuit. Degeneration of the left anterior temporal lobe is associated with linguistic semantic loss, whereas that of the right anterior temporal lobe is associated with prominent behavioral and personality changes, including lack of empathy and increased rigidity (30). Patients with PPA present with focal and asymmetric changes in specific networks fundamental to language processing.

Patients with svPPA present with semantic memory deficit that is localized to the anterior temporal lobes. Abnormalities in white matter connectivity are predominantly distributed over the left fronto-temporal areas, including the uncinate fasciculus, inferior longitudinal fasciculus, corpus callosum, and cingulum (Figure 1; Table 2) (10, 31, 32). The occipital lobe, cerebellum, and brainstem are spared (10, 31). The left temporal lobe variant is approximately three times more common than the right temporal lobe variant (33). Eventually, degeneration spreads to the other side; accordingly, patients with svPPA develop right temporal pole atrophy (and vice versa). The following observations are relatively likely to be associated with major right temporal atrophy and difficulty with person identification (34). Patients with the right temporal variant may have difficulty recognizing famous faces because of semantic loss. As the disease spreads from the temporal lobes into the orbitofrontal cortex, the patients start exhibiting behavioral changes, such as irritability, emotional withdrawal, insomnia, strict or selective eating (often focusing on one particular type of food), and occasionally depression (30). Despite the loss of semantic knowledge in the left temporal lobe variant, functions such as visual attention on the right side are sometimes enhanced. Patients with the left temporal lobe variant are more likely to develop visual compulsions such as repetitions in activities, collecting brightly colored objects, jewelry beading, gardening, or painting (30, 33). By contrast, individuals with the right temporal lobe variant develop verbal compulsions on words and symbols (such as compulsively writing letters, playing solitaire, writing telephone numbers, and making puns). In summary, left temporal atrophy has been associated with loss of verbal semantic knowledge, whereas behavioral symptoms dominate the right temporal variant.

In nfvPPA, the deficits target the frontoinsular cortex. Atrophy is most frequently noted in the left inferior frontal and insular cortices, which disrupts language fluency and grammar (7, 35). White matter abnormalities are predominantly distributed over the left frontal-temporal-parietal regions, including the left superior longitudinal fasciculus, corpus callosum, cingulum, inferior and orbital frontal, anterior temporal, and inferior parietal areas (Figure 1; Table 2) (10, 36). Except for structural MRI, functional MRI, fluorodeoxyglucose (FDG)- PET, and single-photon-emission computed tomography all show disturbances in perfusion and metabolism over these areas (8, 10). In summary, due to cerebral hemispheric differences, patients with right-predominant patterns of atrophy tend to present with behavioral disturbance, such as bvFTD and right-sided svPPA, whereas patients with left-predominant atrophy may lead to language-related impairment, such as left-sided svPPA and nfvPPA.

## Neuropathological Biomarkers: Tau, TDP-43, and Fused in Sarcoma

**Figure 2:**
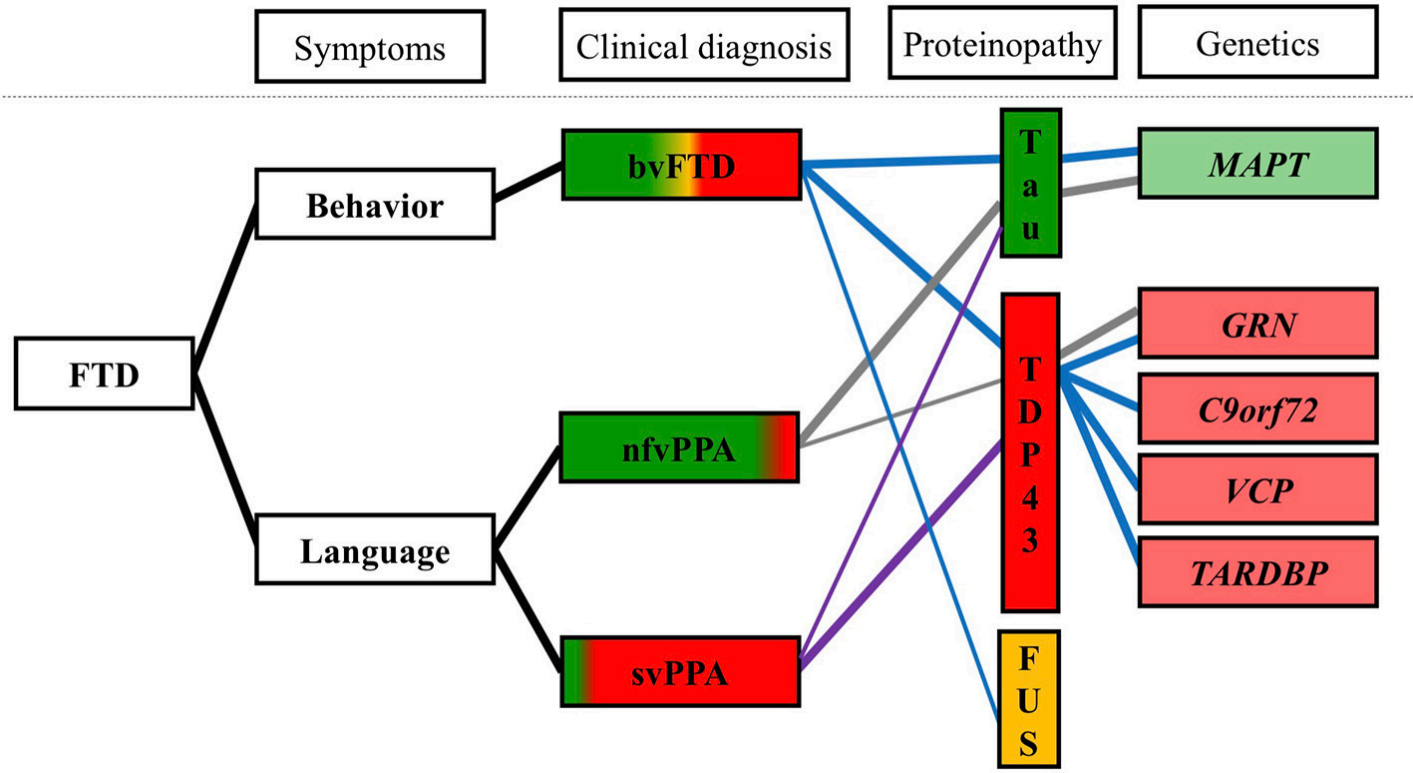
Clinical, pathological, and genetic associations in FTD (7–9, 37). Clinical symptoms are shown at left, colored regions of clinical diagnosis represent relative percentages of patients found to have each underlying neuropathological diagnosis. Blue line, genetic and pathological aspects of bvFTD; gray line, genetic, and pathological aspects of nfvPPA; purple line, genetic, and pathological aspects of svPPA. bvFTD, behavioral-variant frontotemporal dementia; svPPA, semantic-variant primary progressive aphasia; nfvPPA, non-fluent variant primary progressive aphasia; FUS, fused in sarcoma; TDP-43, transactive response (TAR) DNA-binding protein of 43 kDa; *C9orf72*, chromosome 9 open reading frame 72; *GRN*, progranulin mutations; *MAPT*, microtubule-associated protein tau; and *VCP*, valosin-containing protein.

FTD is caused by FTLD, a pathological process of cortical and subcortical degeneration over the frontal and temporal areas. Abnormal intracellular aggregates of tau and transactive response (TAR) DNA-binding protein of 43 kDa (TDP-43) are the leading causes of FTD (accounting for ~90% of cases). Fused in sarcoma (FUS), characterized by abnormal intracellular FUS inclusions, is associated with most of the remaining cases (Figure 2) (7).

### Frontotemporal Lobar Degeneration -Tau Pathology

FTLD-tau accounts for one-third to one-half of all cases of FTLD, characterized by neuronal and glial tau aggregation (8, 38). Tau is critical for cellular morphology and function by binding to and stabilizing microtubules. In neurodegenerative disorders, tau becomes excessively hyperphosphorylated, dissociates from microtubules, and aberrantly aggregates within neurons and glia. Tau is encoded by microtubule-associated protein tau (*MAPT*). *MAPT* mutations mainly result in FTLD-tau pathology (Figure 2). Alternative splicing of *MAPT* mRNA leads to the production of three or four microtubule-binding domain repeats (3R or 4R). FTLD-tau is further subdivided into 3R, 4R, and 3R/4R tauopathies. Pick's disease, a 3R tauopathy, accounts for up to 30% of FTLD-tau cases (8, 38). A striking atrophy over the frontal, cingulate, and temporal gyri was noted (8). CBD, a 4R tauopathy, has been observed in approximately 35% of patients with FTLD-tau and involves the dorsal prefrontal cortex, supplemental motor area, perirolandic cortex, and subcortical nuclei (8, 38). PSP, also a 4R tauopathy, accounts for approximately 30% of patients with FTLD-tau (38). PSP is associated with frontal atrophy and with subcortical atrophy of the globus pallidus, subthalamic nucleus, and brainstem nuclei (8).

Approximately half of all bvFTD patients and the majority of nfvPPA patients have FTLD-tau pathology (Figure 2) (11). Furthermore, in most bvFTD or nfvPPA cases, the presence of extrapyramidal symptoms suggestive of CBS/PSP likely reflect an underlying tauopathy (12). Behavioral changes seen in bvFTD, or non-fluent motor speech difficulties in nfvPPA, may be presented in patients with CBS/PSP clinical syndrome before or after development of movement disorder. PSP neuropathology is strongly associated with patients with PSP clinical syndrome, particularly presence of the supranuclear vertical gaze palsy and early postural instability (11). Thus, PSP patients have become popular for the emergence of tau-targeting therapies in precision medicine. On the other hand, CBS is a highly variable clinical syndrome. In contrast, only 23% of the clinical CBS patients possessed Alzheimer's disease (AD) neuropathology, 13% with PSP neuropathology and 35% with CBD neuropathology in a large series of autopsy (39).

### Frontotemporal Lobar Degeneration-TDP Pathology

FTLD-TDP accounts for approximately half of all patients with FTLD (38). TDP-43, a nuclear protein, is crucial for exon skipping and transcription regulation (40). In FTLD-TDP, TDP-43 becomes aberrantly localized from the nucleus to the cytoplasm, where it forms cytoplasmic inclusions (41). The cytoplasmic inclusions cause neurodegeneration though the potential toxicity of pathological TDP-43 aggregates and loss of normal TDP-43 function (i.e., nuclear clearance, RNA regulation) (11, 42). Four subtypes of FTLD-TDP (types A, B, C, and D) are recognized on the basis of the shape and distribution of TDP-43-positive lesions within the associative cortex (8). Type A accounts for approximately half of nfvPPA patients, one-quarter of suspected CBD patients, and one-quarter of bvFTD patients. Type C accounts for the majority of svPPA patients (Figure 2) (8).

### Frontotemporal Lobar Degeneration -FUS Pathology

FUS is an RNA-binding protein involved in splicing and nuclear export of mRNA. FTLD-FUS has the following three subtypes: atypical FTLD with ubiquitin-positive inclusions, neuronal intermediate filament inclusion disease, and basophilic inclusion body disease (11, 43). A majority of patients with FTLD-FUS pathology are diagnosed as having bvFTD, characterized by sporadic, early-onset FTD with behavioral disturbance, disinhibition, and psychotic symptoms (Figure 2) (44).

Collectively, the diversity of pathology FTLD gives rise to a vast complexity of clinical phenotypes, with often overlapping neuropsychiatric features. Understanding the underlying neuropathological biomarkers, through personalized medicine, may in the future, offer more targeted and precise therapeutic options (Figure 2).

## Genetics Biomarkers

Up to 40% of FTLD patients have a family history of dementia, thus suggesting a familial transmission; however, a clear autosomal-dominant history accounts for only 10% of all patients (45). To date, mutations in *MAPT*, chromosome 9 open reading frame 72 *(C9orf72)*, and progranulin (*GRN*) have accounted for more than half of patients in FTLD families with a strong autosomal-dominant history (8). Mutations in *MAPT* and *GRN* account for 5–20% of patients with familial FTLD; *C9orf72* mutations account for approximately 13–50% of familial FTLD patients and is a common genetic cause of FTD (8, 46, 47).

### MAPT

*MAPT* mutations cause impaired microtubule assembly, impaired axonal transport, and increased pathological tau aggregation (48). As mentioned above, pathological tau formation in the cortical and subcortical brain areas results in neurodegeneration and is neuroanatomically correlated with the development of clinical symptoms (13). Patients with *MAPT* mutations are relatively young at onset (<50 years) and have a relatively short duration of illness (compared with those with other mutations), characterized by psychiatric and behavioral features (i.e., disinhibition, stereotyped repetitive behavior, and obsessions), parkinsonism, and oculomotor dysfunction (13, 49, 50). Neuroimaging studies revealed brain atrophy in the anterior temporal, orbitofrontal, caudate, insula, and anterior cingulate cortices (51), and different *MAPT* mutations may target different brain areas. For example, the medial temporal lobe indicates mutations in the splicing of exon 10, whereas mutations affecting the coding region target the lateral temporal lobe (52).

### C9orf72

The expansion of a noncoding GGGGCC hexanucleotide repeat in *C9orf72* is the most common cause of inherited FTD worldwide, and it accounts for a relatively small proportion of sporadic cases (47, 53). Normally, the number of GGGGCC hexanucleotide repeats is < 20; however, the presence of 65 or more repeats is considered pathogenic. Typically, the number of pathogenic repeats is in the hundreds (54). These expansion mutations are highly associated with TDP-43 pathology (13). FTD patients with the *C9orf72* expansion mutation commonly present with bvFTD, MND, or a combination of the two, characterized by behavioral features (i.e., apathy, loss of empathy, and disinhibition), complex stereotyped behaviors, executive dysfunction, and Parkinson-like symptoms (i.e., gait disturbance, tremor, and rigidity) (13). Approximate 38% of patients present with psychotic symptoms, characterized by delusions, hallucinations, somatic symptoms, agitation, and anxiety (13, 55). Imaging-genetic studies in FTD patients with *C9orf72* expansions have revealed a distributed symmetric pattern of brain atrophy in the frontal lobe (medial, dorsolateral, and orbitofrontal FTD patients with the *C9orf72* expansion may have a more rapid cognitive decline related to cortical atrophy compared with other forms of FTLD-TDP (56, 57), and most *C9orf72* expansion carriers with Parkinson-like symptoms respond poorly to levodopa usage (13). Therefore, *C9orf72* genotyping could potentially be useful for precision medicine approach, not only to classify patients with FTLD but also offer prognostic values.

### GRN

Progranulin is a secreted protein involved in cell-cycle regulation, wound repair, axonal growth, and inflammation modulation (58). *GRN* mutations are associated with haploinsufficiency and the reduction of progranulin production and secretion (59). They are clinically associated with bvFTD, nfvPPA, and CBS, thus conferring relatively low penetrance until 70 years (60, 61). Psychiatric symptoms, including delusions, hallucinations, ritualistic behaviors, apathy, and social withdrawal, are common. Language function is involved early in FTD patients with *GRN* mutations compared with patients with *C9orf72* or *MAPT* mutations (13). Imaging study results showed asymmetric atrophy in the inferior frontal, temporal, and inferior parietal lobes, whereas *C9orf72 or MAPT* mutations are associated with symmetrical brain atrophy (62).

### Other Genetic Biomarkers

Mutations in TAR DNA-binding protein (*TARDBP*), valosin-containing protein (*VCP*), *TIA1, TBK1*, and *CCNF* genes (associated with TDP-43 pathology); and *FUS* and *CHMP2B* (associated with tau-negative, TDP-negative, ubiquitin-positive inclusions) account for a minority of familial FTD (8). Mutations of *TARDBP* are commonly associated with ALS and FTD-ALS, and may be associated with PSP-like symptoms and chorea. Patients with “ALS-plus” symptom (clinical features extending beyond pyramidal and neuromuscular systems) have an increased likelihood of carrying a pathogenic *TARDBP, C9orf72*, or *VCP* mutation in contrast with sporadic cases (63).

Accordingly, genetic assessment for the above known genetic variants may improve diagnosis of FTD amid an overly complicated clinical picture. In addition, it may offer biological information to predict personal disease risk, understand underlying pathophysiology, identify presymptomatic individuals at risk for FTD and even provide future options for personalized therapeutics.

## Applications of FTD Biomarkers for Precision Medicine

Although abnormal tau and TDP protein deposits may not be the only cause of FTD pathogenesis, they can define FTD as a unique neurodegenerative disease in the differential diagnosis of dementia. In addition, the initial differentiation of FTD from atypical AD using FTD precision medicine is crucial because FTD symptoms may become more severe following the application of approved AD therapies (64). A research framework focusing on biomarker diagnosis of FTD is urgently required because of the complex clinicopathological relationships of the disease. With the advent of biomarkers diagnostic techniques such as neuroimaging, biofluid dynamics, and genetics, FTD pathology can be identified; this can greatly improve diagnostic precision regarding the underlying pathophysiology of clinical syndromes. The National Institute on Aging and Alzheimer's Association recently introduced a new research framework for AD diagnosis based on biomarkers (65). A future diagnostic framework for FTD could adopt a similar approach for precision medicine. The best neuroimaging approach to separate AD from FTD is by FDG-PET, as evident by the diffuse posterior temporoparietal hypometabolism in AD vs. the focal frontotemporal hypometabolism in FTD (8, 10). Although a high proportion of FTLD patients have low levels of comorbid AD neuropathology and amyloid-beta (Aβ_1−42_) imaging is non-specific, amyloid PET scanning may still be useful for such differentiation (11). However, total tau (t-tau) and Aβ_1−42_ are extensively studied cerebrospinal fluid (CSF) biomarkers that may be used to accurately distinguish autopsy-confirmed FTD from AD (66); FTD patients have lower t-tau–Aβ_1−42_ ratios (37). The CSF t-tau–Aβ_1−42_ ratio may lead to substantial improvement in clinical diagnosis for differentiating FTD from atypical AD (67). Levels of CSF biomarkers of axonal injury and neuronal loss such as neurofilament light chains are reportedly elevated in clinical FTD cohorts compared with cohorts of other neurodegenerative diseases (68, 69) and have been associated with FTD disease severity (69).

A key task is to distinguish FTD from transmissible spongiform encephalopathies [i.e., Creutzfeldt-Jakob disease (CJD)], especially when early symptoms are subtle. CJD is a typical human prion disease caused by the aggregation and propagation of scrapie prion protein (PrP^SC^)—a misfolded form of normal prion protein (PrP^C^). CJD has many forms, including familial, variant, iatrogenic, and sporadic. Sporadic is the most common form (appropriately 85%) (70). Symptoms include myoclonus, rapidly progressive dementia, pyramidal/extrapyramidal signs, visual/cerebellar symptoms, and akinetic mutism. Clinical diagnosis of sporadic CJD (sCJD) is supported by the identification of 14-3-3 protein in CSF, periodic sharp electroencephalographic spikes, or MRI T2 FLAIR or diffusion-weighted imaging hyperintensities in the basal ganglia and cerebral or cerebellar cortex (71, 72). Compared with FTD, in sCJD, the CSF t-tau level is higher and the p-tau-t-tau ratio is lower (73). The aforementioned clinical, imaging, and biofluid markers are helpful in discriminating patients with FTD from those with CJD when clinical images are early and subtle.

Based on protein-targeting therapies such as those targeting tau (74), differentiation of FTLD-tau from FTLD-TDP after the exclusion of other neurodegenerative diseases (i.e., atypical AD and CJD) is the third step. Compared with FTLD-TDP and ALS, FTLD-tau has a higher p-tau level and higher p-tau-t-tau ratio (11). FTLD-TDP does not have significant p-tau pathology, and thus less p-tau may be released into the CSF compared with FTLD-tau (68, 75). The quantitative immunoprecipitation approach has been developed to detect specific forms of tau [extended (55 kDa) and truncated (33 kDa)] (76) to differentiate FTLD-tau from its subtypes. Through the exploratory proteomics-based approach, many novel CSF biomarkers have been identified for discriminating FTLD-tau from the main FTLD pathological subtypes as well as from non-demented controls and other forms of dementia with maximal accuracy (77). Studies to demonstrate the use of non-invasive methods such as the application of tau-specific radio ligands to identify FTLD-tau cases are ongoing (78, 79). Imaging and CSF biomarkers may assist in the development of successful therapies by facilitating the appropriate selection of cases for clinical trials targeting specific proteinopathies.

The longitudinal progression of biomarkers at early disease stages may be understood through the investigation of presymptomatic individuals within families that possess pathogenic mutations such as an earlier age of FTLD-tau onset with *MAPT* mutations (64). Brain network dysfunction has been observed in presymptomatic FTD with *GRN* (80) and *C9orf72* (81) mutations. *C9orf72* disease exhibits additional protein inclusion, additional clinical symptoms, and worse prognosis compared with its sporadic forms (11).

In line with the popular use of the *APOE* genotype in AD clinical trials, hereditary FTLD may be a popular choice for clinical trial development of therapies specific to this mutation. An autopsy-confirmed sporadic FTLD genetic study revealed several single-nucleotide polymorphisms (SNPs) that were overexpressed in patients with FTLD-tau and those with FTLD-TDP (82). The risk allele in the FTLD-tau-related SNP was associated with a shorter disease duration and white matter loss in the midbrain and long association fibers in sporadic bvFTD (83). This indicates the usefulness of SNP genotyping as a diagnostic and prognostic tool. A syndrome constitutes a clinical outcome of one or multiple diseases as opposed to an etiology. Similar to AD, a biological definition of FTD as opposed to a syndromal one is a superior means of enhancing the understanding of the underlying mechanisms of the clinical expression of FTD (65). Future precision-medicine approaches for FTD treatment must include biologically defined targets alongside the establishment of biomarker profiles and categories.

## Treatments

Nonpharmacological interventions are considered for the management of dementia before the use of pharmacological treatments that may exacerbate medical comorbidities affecting elderly patients. Healthy lifestyle changes, social connections, physical activity, and environmental intervention may mitigate the effects of dementia (42, 43). The main purpose of nonpharmacological interventions is to prevent disruptive behaviors, provide symptom remission, and reduce caregiver distress. For example, environmental approaches (i.e., reduction of noise, limitation of stimuli, and simplification of daily activities and social parameters) intend to reduce irritability, aggression, and anxiety caused by daily external stimuli.

Currently, no disease-modifying drugs approved by the U.S. Food and Drug Administration are available for the treatment of FTD. Most treatments are focused on the management of behavioral symptoms. The use of selective serotonin reuptake inhibitors can reduce the severity of agitation, aggressiveness, impulsivity, aberrant eating behaviors, and compulsions (8). Herrmann et al. reported that behavioral and psychiatric symptoms (including irritability, disinhibition, and depression) were alleviated after citalopram treatment at a target dose of 40 mg once daily; furthermore, they observed a decrease in the overall Neuropsychiatric Inventory and Frontal Behavioral Inventory scores (84). The findings suggest that antidepressants may be beneficial in the treatment of neuropsychiatric and behavioral disturbances in FTD.

Behavioral disturbance (agitation or impulsivity) may also be controlled using atypical antipsychotics such as risperidone, olanzapine, and quetiapine. However, these medications could have side effects and increase the risk of mortality in patients with FTD (13, 85). In FTD patients with *C9orf72* expansion, antipsychotic drugs cause noticeable adverse effects, and the adverse effects could not be reversed in some patients even after drug withdrawal (86, 87). In summary, low doses of atypical antipsychotic drugs may be useful for managing behavioral disturbance (8), but such drugs should be used with caution in patients with FTD because of the risk of mortality associated with cardiac events and falls secondary to the side effects (88).

Cholinesterase inhibitors, such as donepezil, do not alleviate but can even exacerbate behavioral disturbance in patients with FTD (89). This is likely because cholinergic deficit may not contribute to the pathophysiology of FTD (90). Memantine, an *N*-methyl-d-aspartate (NMDA) antagonist, has been reported to have no benefits in alleviating or delaying the progression of FTD symptoms, but it is generally well-tolerated by patients (91, 92). In summary, cholinesterase inhibitors and NMDA antagonist have no clinical efficacy in the treatment of FTD, and they potentially have detrimental effects on cognitive performance and behavioral symptoms (91, 93). Therefore, preferred management options should be based on risk assessment, and nonpharmacological interventions and caregiver support are preferred for first-line interventions (93).

## Tau-Targeting Therapeutics

Due to knowledge advancements in molecular biology, pathophysiology, and neuropathology, precision medicine could be applied in FTD treatment by targeting the underlying pathogenesis (85). Based on the knowledge of the pathological tau protein spreading through prion-like propagation, the prevention of transneuronal spreading of pathological abnormalities is potentially an effective therapeutic strategy (94, 95). Administering tau aggregation inhibitors (i.e., methylthioninium chloride), microtubule-stabilizing drugs, tau-targeted immunotherapy, and tau vaccines may be useful therapeutic approaches in patients with tau pathology (96, 97).

Multiple approaches are available for tau-targeting therapeutics. First, tau aggregation and the various tau species formed (monomers, oligomers, prefilaments, granules, fibrils, and insoluble aggregates) during aggregation are of interest for potential therapeutic intervention. Hence, tau aggregation inhibitors have been proven effective in various *in vitro* studies (98). A proprietary formulation of non-neuroleptic phenothiazine methylene blue (methylthioninium chloride), which is used to treat malaria (99), has risen in the ranks in clinical development in recent years. This compound readily crosses the blood-brain barrier and prevents tau aggregation *in vitro* as well as in cell and animal models (100, 101). Safety and efficacy in a randomized, double-blind, placebo-controlled, multinational, and parallel-group clinical trial was demonstrated in 220 patients with bvFTD after 12 months of oral treatment; the results are yet to be published (74). Clarifying the efficacy of tau aggregation inhibitors *in vivo* is critical for preventing cognitive decline.

Second, microtubule stabilizers may be used as FTLD-tau therapeutic agents. Detachment of tau from microtubules leads to the loss of normal microtubule-stabilizing function, resulting in axonal transport defects and synaptic dysfunction. Davunetide—an eight-amino-acid peptide that arises from an activity-dependent neuroprotective protein-exerted substantial effects on behavior and cognition in tau-transgenic mice (102). In addition, intranasal or intravenous administration of davunetide established the safety and tolerability profile of davunetide in patients with mild cognitive impairment (103). However, whether microtubule destabilization is directly related to tau toxicity in tauopathies remains unclear. No therapeutic effect of davunetide for PSP treatment was detected in a double-blind, placebo-controlled, randomized phase II/III clinical trial (104).

Third, various anti-tau immunotherapy strategies have been successfully tested, suggesting that such strategies could be feasible options for clearing toxic protein species in tauopathies (105). Targeting abnormally phosphorylated tau epitopes (or pathologically relevant conformational epitopes) may be favorable for inducing antibody responses that promote tau clearance, as suggested by evidence found in animal models (106). The humanized anti-tau monoclonal antibody named ABBV-8E12 is also available for PSP treatment. A satisfactory safety and tolerability profile for ABBV-8E12 was demonstrated in a placebo-controlled, double-blind, phase I single-ascending-dose trial of 30 patients with PSP (107). Finally, modulating tau phosphorylation and targeting other posttranslational tau modifications (i.e., tau acetylation inhibitors) are also potential therapeutic strategies for FTLD-tau and other tauopathies (74). However, the current consensus is that tau-centric targeted treatment has no marked effect on long-term clinical outcomes (108, 109); further research is required to elucidate the potential roles of such therapies in treating FTD.

Other targets for therapy include disrupting the downstream effects of *C9orf72* and *GRN* mutations. An approach that entails developing antisense oligonucleotides to reduce the concentrations of potentially toxic *C9orf72* mRNAs has been applied to FTD patients (110, 111). This approach has also been implicated in the reduction of the total amount of pathological tau species (112). Because *GRN* mutation is related to progranulin haploinsufficiency and reduced progranulin concentrations, studies are attempting to adopt molecular approaches to prevent the reduction of progranulin concentrations and instead increase progranulin concentrations; such approaches include applying the histone deacetylase inhibitor suberoylanilide hydroxamic acid, which enhances progranulin transcription and alkalizing compounds that stimulate progranulin production (113, 114). Collectively, advances in the understanding of genetic mutations causing FTD have created new potential therapeutic targets for the development of effective disease-modifying drugs (85). These findings may enable physicians to develop precision medicine for the treatment of FTD patients with *C9orf72, MAPT, or GRN* mutations at a single-patient levels (10, 115).

### Future Direction- Treatment Through Personalized Medicine

The implication of precision medicine is to enable physicians to identify highly selective and effective treatments with relatively few side effects for patients with specific illness. Currently, precision medicine is applied to FTD diagnosis and treatment. Based on advances in neuroimaging and genomic research that has explored underlying genetic risk variants and cerebral structural and functional change in order to determine specific molecular pathways and pathophysiological processes, precision medicine is currently applied in clinical trials; such trials focus on subgroups of individuals and the development of therapeutic targets with known genetic risk for FTD (116). For example, in patients with clinical diagnosis of bvFTD, tau-targeting therapeutics (e.g., tau aggregation inhibitors) may be administered in those with suspected FTLD-tau pathology. On the other hand, in bvFTD patients with suspected FTLD-TDP pathology, progranulin -related therapies may be a viable treatment for those with *GRN* mutation. Likewise, for those with *C9orf72* repeat expansions, candidate antisense therapeutics could be used to reduce C9ORF72 expression. However, this is only the beginning of the precision medicine approach targeting the clinical process and treatment response of FTD. Collaboration among parents, family caregivers, and professionals (e.g., clinicians, scientists, and medical technologists) is crucial for identifying the pathological processes underlying FTD and for developing new interventions for successful application of precision medicine.

## Author Contributions

All authors listed have made a substantial, direct and intellectual contribution to the work, and approved it for publication.

### Conflict of Interest Statement

The authors declare that the research was conducted in the absence of any commercial or financial relationships that could be construed as a potential conflict of interest.

## References

[1] Vieira RT Caixeta L Machado S Silva AC Nardi AE Arias-Carrion O. Epidemiology of early-onset dementia: a review of the literature. Clin Pract Epidemiol Ment Health (2013) 9:88–95. 10.2174/1745017901309010088 23878613 PMC3715758

[2] Pick A Über die Beziehungen der senilen Hirnatrophie zur Aphasie. Prager Med Wochenschr. (1892) 17:165–67.

[3] Alzheimer A Über eigenartige Krankheitsfälle der späteren Alters. Z Gesamte Neurol Psychiatr. (1911) 4:356–85.

[4] Mesulam MM Primary progressive aphasia. Ann Neurol. (2001) 49:425–32. 10.1002/ana.91 11310619

[5] Rascovsky K Hodges JR Knopman D Mendez MF Kramer JH Neuhaus J. Sensitivity of revised diagnostic criteria for the behavioural variant of frontotemporal dementia. Brain (2011) 134(Pt 9):2456–77. 10.1093/brain/awr179 21810890 PMC3170532

[6] Gorno-Tempini ML Hillis AE Weintraub S Kertesz A Mendez M Cappa SF. Classification of primary progressive aphasia and its variants. Neurology (2011) 76:1006–14. 10.1212/WNL.0b013e31821103e6 21325651 PMC3059138

[7] Elahi FM Miller BL. A clinicopathological approach to the diagnosis of dementia. Nat Rev Neurol. (2017) 13:457–76. 10.1038/nrneurol.2017.96 28708131 PMC5771416

[8] Bang J Spina S Miller BL. Frontotemporal dementia. Lancet (2015) 386:1672–82. 10.1016/S0140-6736(15)00461-4 26595641 PMC5970949

[9] Olney NT Spina S Miller BL. Frontotemporal dementia. Neurol Clin. (2017) 35:339–74. 10.1016/j.ncl.2017.01.008 28410663 PMC5472209

[10] Gordon E Rohrer JD Fox NC. Advances in neuroimaging in frontotemporal dementia. J Neurochem. (2016) 138(Suppl. 1):193–210. 10.1111/jnc.13656 27502125

[11] Irwin DJ Cairns NJ Grossman M McMillan CT Lee EB Van Deerlin VM. Frontotemporal lobar degeneration: defining phenotypic diversity through personalized medicine. Acta Neuropathol. (2015) 129:469–91. 10.1007/s00401-014-1380-1 25549971 PMC4369168

[12] Forman MS Farmer J Johnson JK Clark CM Arnold SE Coslett HB. Frontotemporal dementia: clinicopathological correlations. Ann Neurol. (2006) 59:952–62. 10.1002/ana.20873 16718704 PMC2629792

[13] Young JJ Lavakumar M Tampi D Balachandran S Tampi RR. Frontotemporal dementia: latest evidence and clinical implications. Ther Adv Psychopharmacol. (2018) 8:33–48. 10.1177/2045125317739818 29344342 PMC5761910

[14] Rogalski E Cobia D Martersteck A Rademaker A Wieneke C Weintraub S. Asymmetry of cortical decline in subtypes of primary progressive aphasia. Neurology (2014) 83:1184–91. 10.1212/WNL.0000000000000824 25165386 PMC4176026

[15] Garcin B Lillo P Hornberger M Piguet O Dawson K Nestor PJ. Determinants of survival in behavioral variant frontotemporal dementia. Neurology (2009) 73:1656–61. 10.1212/WNL.0b013e3181c1dee7 19917988 PMC2881857

[16] Rankin KP Santos-Modesitt W Kramer JH Pavlic D Beckman V Miller BL. Spontaneous social behaviors discriminate behavioral dementias from psychiatric disorders and other dementias. J Clin Psychiatry (2008) 69:60–73. 10.4088/JCP.v69n0109 18312039 PMC2735556

[17] Velakoulis D Walterfang M Mocellin R Pantelis C McLean C. Frontotemporal dementia presenting as schizophrenia-like psychosis in young people: clinicopathological series and review of cases. Br J Psychiatry (2009) 194:298–305. 10.1192/bjp.bp.108.057034 19336778

[18] Sha SJ Takada LT Rankin KP Yokoyama JS Rutherford NJ Fong JC. Frontotemporal dementia due to C9ORF72 mutations: clinical and imaging features. Neurology (2012) 79:1002–11. 10.1212/WNL.0b013e318268452e 22875087 PMC3430713

[19] Gorno-Tempini ML Brambati SM Ginex V Ogar J Dronkers NF Marcone A. The logopenic/phonological variant of primary progressive aphasia. Neurology (2008) 71:1227–34. 10.1212/01.wnl.0000320506.79811.da 18633132 PMC2676989

[20] Kramer JH Jurik J Sha SJ Rankin KP Rosen HJ Johnson JK. Distinctive neuropsychological patterns in frontotemporal dementia, semantic dementia, and Alzheimer disease. Cogn Behav Neurol. (2003) 16:211–8. 10.1097/00146965-200312000-00002 14665820

[21] Josephs KA Duffy JR Strand EA Whitwell JL Layton KF Parisi JE. Clinicopathological and imaging correlates of progressive aphasia and apraxia of speech. Brain (2006) 129(Pt 6):1385–98. 10.1093/brain/awl078 16613895 PMC2748312

[22] Burrell JR Kiernan MC Vucic S Hodges JR. Motor neuron dysfunction in frontotemporal dementia. Brain (2011) 134(Pt 9):2582–94. 10.1093/brain/awr195 21840887

[23] Le Ber I Guedj E Gabelle A Verpillat P Volteau M Thomas-Anterion C. Demographic, neurological and behavioural characteristics and brain perfusion SPECT in frontal variant of frontotemporal dementia. Brain (2006) 129(Pt 11):3051–65. 10.1093/brain/awl288 17071924

[24] Armstrong MJ Litvan I Lang AE Bak TH Bhatia KP Borroni B. Criteria for the diagnosis of corticobasal degeneration. Neurology (2013) 80:496–503. 10.1212/WNL.0b013e31827f0fd1 23359374 PMC3590050

[25] Hoglinger GU Respondek G Stamelou M Kurz C Josephs KA Lang AE. Clinical diagnosis of progressive supranuclear palsy: the movement disorder society criteria. Mov Disord. (2017) 32:853–64. 10.1002/mds.26987 28467028 PMC5516529

[26] Litvan I Agid Y Calne D Campbell G Dubois B Duvoisin RC. Clinical research criteria for the diagnosis of progressive supranuclear palsy (Steele-Richardson-Olszewski syndrome): report of the NINDS-SPSP international workshop. Neurology (1996) 47:1–9. 10.1212/WNL.47.1.1 8710059

[27] Rosen HJ Allison SC Schauer GF Gorno-Tempini ML Weiner MW Miller BL. Neuroanatomical correlates of behavioural disorders in dementia. Brain (2005) 128(Pt 11):2612–25. 10.1093/brain/awh628 16195246 PMC1820861

[28] Seeley WW Crawford R Rascovsky K Kramer JH Weiner M Miller BL. Frontal paralimbic network atrophy in very mild behavioral variant frontotemporal dementia. Arch Neurol. (2008) 65:249–55. 10.1001/archneurol.2007.38 18268196 PMC2544627

[29] Diehl-Schmid J Onur OA Kuhn J Gruppe T Drzezga A. Imaging frontotemporal lobar degeneration. Curr Neurol Neurosci Rep. (2014) 14:489. 10.1007/s11910-014-0489-x 25171901

[30] Seeley WW Bauer AM Miller BL Gorno-Tempini ML Kramer JH Weiner M. The natural history of temporal variant frontotemporal dementia. Neurology (2005) 64:1384–90. 10.1212/01.WNL.0000158425.46019.5C 15851728 PMC2376750

[31] Lam BY Halliday GM Irish M Hodges JR Piguet O. Longitudinal white matter changes in frontotemporal dementia subtypes. Hum Brain Mapp. (2014) 35:3547–57. 10.1002/hbm.22420 25050433 PMC6869363

[32] Tu S Leyton CE Hodges JR Piguet O Hornberger M. Divergent longitudinal propagation of white matter degradation in logopenic and semantic variants of primary progressive aphasia. J Alzheimers Dis. (2016) 49:853–61. 10.3233/JAD-150626 26484929

[33] Thompson SA Patterson K Hodges JR. Left/right asymmetry of atrophy in semantic dementia: behavioral-cognitive implications. Neurology (2003) 61:1196–203. 10.1212/01.WNL.0000091868.28557.B8 14610120

[34] Chan D Anderson V Pijnenburg Y Whitwell J Barnes J Scahill R. The clinical profile of right temporal lobe atrophy. Brain (2009) 132(Pt 5):1287–98. 10.1093/brain/awp037 19297506

[35] Gorno-Tempini ML Dronkers NF Rankin KP Ogar JM Phengrasamy L Rosen HJ. Cognition and anatomy in three variants of primary progressive aphasia. Ann Neurol. (2004) 55:335–46. 10.1002/ana.10825 14991811 PMC2362399

[36] Galantucci S Tartaglia MC Wilson SM Henry ML Filippi M Agosta F. White matter damage in primary progressive aphasias: a diffusion tensor tractography study. Brain (2011) 134(Pt 10):3011–29. 10.1093/brain/awr099 21666264 PMC3187537

[37] Struyfs H Niemantsverdriet E Goossens J Fransen E Martin JJ De Deyn PP. Cerebrospinal fluid P-Tau181P: biomarker for improved differential dementia diagnosis. Front Neurol. (2015) 6:138. 10.3389/fneur.2015.00138 26136723 PMC4470274

[38] Josephs KA Hodges JR Snowden JS Mackenzie IR Neumann M Mann DM. Neuropathological background of phenotypical variability in frontotemporal dementia. Acta Neuropathol. (2011) 122:137–53. 10.1007/s00401-011-0839-6 21614463 PMC3232515

[39] Lee SE Rabinovici GD Mayo MC Wilson SM Seeley WW DeArmond SJ. Clinicopathological correlations in corticobasal degeneration. Ann Neurol. (2011) 70:327–40. 10.1002/ana.22424 21823158 PMC3154081

[40] Bhardwaj A Myers MP Buratti E Baralle FE. Characterizing TDP-43 interaction with its RNA targets. Nucleic Acids Res. (2013) 41:5062–74. 10.1093/nar/gkt189 23519609 PMC3643599

[41] Neumann M Sampathu DM Kwong LK Truax AC Micsenyi MC Chou TT. Ubiquitinated TDP-43 in frontotemporal lobar degeneration and amyotrophic lateral sclerosis. Science (2006) 314:130–3. 10.1126/science.1134108 17023659

[42] Lee EB Lee VM Trojanowski JQ. Gains or losses: molecular mechanisms of TDP43-mediated neurodegeneration. Nat Rev Neurosci. (2011) 13:38–50. 10.1038/nrn3121 22127299 PMC3285250

[43] Mackenzie IR Munoz DG Kusaka H Yokota O Ishihara K Roeber S. Distinct pathological subtypes of FTLD-FUS. Acta Neuropathol. (2011) 121:207–18. 10.1007/s00401-010-0764-0 21052700

[44] Riedl L Mackenzie IR Forstl H Kurz A Diehl-Schmid J. Frontotemporal lobar degeneration: current perspectives. Neuropsychiatr Dis Treat. (2014) 10:297–310. 10.2147/NDT.S38706 24600223 PMC3928059

[45] Rohrer JD Guerreiro R Vandrovcova J Uphill J Reiman D Beck J. The heritability and genetics of frontotemporal lobar degeneration. Neurology (2009) 73:1451–6. 10.1212/WNL.0b013e3181bf997a 19884572 PMC2779007

[46] Rademakers R Neumann M Mackenzie IR. Advances in understanding the molecular basis of frontotemporal dementia. Nat Rev Neurol. (2012) 8:423–34. 10.1038/nrneurol.2012.117 22732773 PMC3629543

[47] DeJesus-Hernandez M Mackenzie IR Boeve BF Boxer AL Baker M Rutherford NJ. Expanded GGGGCC hexanucleotide repeat in noncoding region of C9ORF72 causes chromosome 9p-linked FTD and ALS. Neuron (2011) 72:245–56. 10.1016/j.neuron.2011.09.011 21944778 PMC3202986

[48] Hutton M Lendon CL Rizzu P Baker M Froelich S Houlden H. Association of missense and 5'-splice-site mutations in tau with the inherited dementia FTDP-17. Nature (1998) 393:702–5. 10.1038/31508 9641683

[49] Ng AS. Genetics of frontotemporal dementia in Asia: advancing knowledge through collaboration. Neurology (2015) 85:2060–2. 10.1212/WNL.0000000000002045 26432849

[50] Boeve BF Hutton M. Refining frontotemporal dementia with parkinsonism linked to chromosome 17: introducing FTDP-17 (MAPT) and FTDP-17 (PGRN). Arch Neurol. (2008) 65:460–4. 10.1001/archneur.65.4.460 18413467 PMC2746630

[51] Ghetti B Oblak AL Boeve BF Johnson KA Dickerson BC Goedert M. Invited review: frontotemporal dementia caused by microtubule-associated protein tau gene (MAPT) mutations: a chameleon for neuropathology and neuroimaging. Neuropathol Appl Neurobiol. (2015) 41:24–46. 10.1111/nan.12213 25556536 PMC4329416

[52] Whitwell JL Jack CR Jr Boeve BF Senjem ML Baker M Ivnik RJ. Atrophy patterns in IVS10+16, IVS10+3, N279K, S305N, P301L, and V337M MAPT mutations. Neurology (2009) 73:1058–65. 10.1212/WNL.0b013e3181b9c8b9 19786698 PMC2754325

[53] Renton AE Majounie E Waite A Simon-Sanchez J Rollinson S Gibbs JR. A hexanucleotide repeat expansion in C9ORF72 is the cause of chromosome 9p21-linked ALS-FTD. Neuron (2011) 72:257–68. 10.1016/j.neuron.2011.09.010 21944779 PMC3200438

[54] Loy CT Schofield PR Turner AM Kwok JB. Genetics of dementia. Lancet (2014) 383:828–40. 10.1016/S0140-6736(13)60630-3 23927914

[55] Snowden JS Rollinson S Thompson JC Harris JM Stopford CL Richardson AM. Distinct clinical and pathological characteristics of frontotemporal dementia associated with C9ORF72 mutations. Brain (2012) 135(Pt 3):693–708. 10.1093/brain/awr355 22300873 PMC3286329

[56] Floeter MK Gendron TF. Biomarkers for amyotrophic lateral sclerosis and frontotemporal dementia associated with hexanucleotide expansion mutations in C9orf72. Front Neurol. (2018) 9:1063. 10.3389/fneur.2018.01063 30568632 PMC6289985

[57] Irwin DJ McMillan CT Brettschneider J Libon DJ Powers J Rascovsky K. Cognitive decline and reduced survival in C9orf72 expansion frontotemporal degeneration and amyotrophic lateral sclerosis. J Neurol Neurosurg Psychiatry (2013) 84:163–9. 10.1136/jnnp-2012-303507 23117491 PMC3543474

[58] Toh H Chitramuthu BP Bennett HP Bateman A. Structure, function, and mechanism of progranulin; the brain and beyond. J Mol Neurosci. (2011) 45:538–48. 10.1007/s12031-011-9569-4 21691802

[59] Shankaran SS Capell A Hruscha AT Fellerer K Neumann M Schmid B. Missense mutations in the progranulin gene linked to frontotemporal lobar degeneration with ubiquitin-immunoreactive inclusions reduce progranulin production and secretion. J Biol Chem. (2008) 283:1744–53. 10.1074/jbc.M705115200 17984093

[60] Gass J Cannon A Mackenzie IR Boeve B Baker M Adamson J. Mutations in progranulin are a major cause of ubiquitin-positive frontotemporal lobar degeneration. Hum Mol Genet. (2006) 15:2988–3001. 10.1093/hmg/ddl241 16950801

[61] Mackenzie IR Neumann M. Molecular neuropathology of frontotemporal dementia: insights into disease mechanisms from postmortem studies. J Neurochem. (2016) 138 (Suppl 1):54–70. 10.1111/jnc.13588 27306735

[62] Rohrer JD Ridgway GR Modat M Ourselin S Mead S Fox NC. Distinct profiles of brain atrophy in frontotemporal lobar degeneration caused by progranulin and tau mutations. Neuroimage (2010) 53:1070–6. 10.1016/j.neuroimage.2009.12.088 20045477 PMC2941039

[63] McCluskey L Vandriel S Elman L Van Deerlin VM Powers J Boller A. ALS-Plus syndrome: non-pyramidal features in a large ALS cohort. J Neurol Sci. (2014) 345:118–24. 10.1016/j.jns.2014.07.022 25086858 PMC4177937

[64] Boxer AL Gold M Huey E Hu WT Rosen H Kramer J. The advantages of frontotemporal degeneration drug development (part 2 of frontotemporal degeneration: the next therapeutic frontier). Alzheimers Dement. (2013) 9:189–98. 10.1016/j.jalz.2012.03.003 23062850 PMC3562382

[65] Jack CR Jr Bennett DA Blennow K Carrillo MC Dunn B Haeberlein SB. NIA-AA research framework: toward a biological definition of Alzheimer's disease. Alzheimers Dement. (2018) 14:535–62. 10.1016/j.jalz.2018.02.018 29653606 PMC5958625

[66] Irwin DJ Trojanowski JQ Grossman M. Cerebrospinal fluid biomarkers for differentiation of frontotemporal lobar degeneration from Alzheimer's disease. Front Aging Neurosci. (2013) 5:6. 10.3389/fnagi.2013.00006 23440936 PMC3578350

[67] Irwin DJ McMillan CT Toledo JB Arnold SE Shaw LM Wang LS. Comparison of cerebrospinal fluid levels of tau and Abeta 1-42 in Alzheimer disease and frontotemporal degeneration using 2 analytical platforms. Arch Neurol. (2012) 69:1018–25. 10.1001/archneurol.2012.26 22490326 PMC3528180

[68] Kortvelyessy P Heinze HJ Prudlo J Bittner D. CSF biomarkers of neurodegeneration in progressive non-fluent aphasia and other forms of frontotemporal dementia: clues for pathomechanisms? Front Neurol. (2018) 9:504. 10.3389/fneur.2018.00504 30013506 PMC6036143

[69] Scherling CS Hall T Berisha F Klepac K Karydas A Coppola G. Cerebrospinal fluid neurofilament concentration reflects disease severity in frontotemporal degeneration. Ann Neurol. (2014) 75:116–26. 10.1002/ana.24052 24242746 PMC4020786

[70] Danev SI St Stoyanov D. Early noninvasive diagnosis of neurodegenerative diseases. Folia Med. (2010) 52:5–13. 10.2478/v10153-010-0041-y 20836391

[71] Zerr I Kallenberg K Summers DM Romero C Taratuto A Heinemann U. Updated clinical diagnostic criteria for sporadic Creutzfeldt-Jakob disease. Brain (2009) 132(Pt 10):2659–68. 10.1093/brain/awp191 19773352 PMC2759336

[72] Vitali P Maccagnano E Caverzasi E Henry RG Haman A Torres-Chae C. Diffusion-weighted MRI hyperintensity patterns differentiate CJD from other rapid dementias. Neurology (2011) 76:1711–9. 10.1212/WNL.0b013e31821a4439 21471469 PMC3100134

[73] Riemenschneider M Wagenpfeil S Vanderstichele H Otto M Wiltfang J Kretzschmar H. Phospho-tau/total tau ratio in cerebrospinal fluid discriminates Creutzfeldt-Jakob disease from other dementias. Mol Psychiatry (2003) 8:343–7. 10.1038/sj.mp.4001220 12660807

[74] Medina M. An Overview on the clinical development of tau-based therapeutics. Int J Mol Sci. (2018) 19:E1160. 10.3390/ijms19041160 29641484 PMC5979300

[75] Hu WT Watts K Grossman M Glass J Lah JJ Hales C. Reduced CSF p-Tau181 to Tau ratio is a biomarker for FTLD-TDP. Neurology (2013) 81:1945–52. 10.1212/01.wnl.0000436625.63650.27 24174584 PMC3843382

[76] Borroni B Gardoni F Parnetti L Magno L Malinverno M Saggese E. Pattern of Tau forms in CSF is altered in progressive supranuclear palsy. Neurobiol Aging (2009) 30:34–40. 10.1016/j.neurobiolaging.2007.05.009 17709155

[77] Teunissen CE Elias N Koel-Simmelink MJ Durieux-Lu S Malekzadeh A Pham TV. Novel diagnostic cerebrospinal fluid biomarkers for pathologic subtypes of frontotemporal dementia identified by proteomics. Alzheimers Dement. (2016) 2:86–94. 10.1016/j.dadm.2015.12.004 27239539 PMC4879654

[78] Brendel M Yousefi BH Blume T Herz M Focke C Deussing M Comparison of F-T807 and F-THK5117 PET in a mouse model of tau pathology. Front Aging Neurosci. (2018) 10:174 10.3389/fnagi.2018.00174 29930508 PMC5999706

[79] Goedert M Yamaguchi Y Mishra SK Higuchi M Sahara N. Tau Filaments and the development of positron emission tomography Tracers. Front Neurol. (2018) 9:70. 10.3389/fneur.2018.00070 29497399 PMC5818396

[80] Dopper EG Rombouts SA Jiskoot LC den Heijer T de Graaf JR de Koning I. Structural and functional brain connectivity in presymptomatic familial frontotemporal dementia. Neurology (2014) 83:e19–26. 10.1212/WNL.0000000000000583 25002573

[81] Lee SE Khazenzon AM Trujillo AJ Guo CC Yokoyama JS Sha SJ. Altered network connectivity in frontotemporal dementia with C9orf72 hexanucleotide repeat expansion. Brain (2014) 137(Pt 11):3047–60. 10.1093/brain/awu248 25273996 PMC4208465

[82] McMillan CT Toledo JB Avants BB Cook PA Wood EM Suh E. Genetic and neuroanatomic associations in sporadic frontotemporal lobar degeneration. Neurobiol Aging (2014) 35:1473–82. 10.1016/j.neurobiolaging.2013.11.029 24373676 PMC3961542

[83] Irwin DJ McMillan CT Suh E Powers J Rascovsky K Wood EM. Myelin oligodendrocyte basic protein and prognosis in behavioral-variant frontotemporal dementia. Neurology (2014) 83:502–9. 10.1212/WNL.0000000000000668 24994843 PMC4141997

[84] Herrmann N Black SE Chow T Cappell J Tang-Wai DF Lanctot KL. Serotonergic function and treatment of behavioral and psychological symptoms of frontotemporal dementia. Am J Geriatr Psychiatry (2012) 20:789–97. 10.1097/JGP.0b013e31823033f3 21878805

[85] Tsai RM Boxer AL. Therapy and clinical trials in frontotemporal dementia: past, present, and future. J Neurochem. (2016) 138 (Suppl. 1):211–21. 10.1111/jnc.13640 27306957 PMC5217534

[86] Landqvist Waldo M Gustafson L Nilsson K Traynor BJ Renton AE Englund E. Frontotemporal dementia with a C9ORF72 expansion in a Swedish family: clinical and neuropathological characteristics. Am J Neurodegener Dis. (2013) 2:276–86. 24319645 PMC3852567

[87] Ducharme S Bajestan S Dickerson BC Voon V. Psychiatric presentations of C9orf72 mutation: what are the diagnostic implications for clinicians? J Neuropsychiatry Clin Neurosci. (2017) 29:195–205. 10.1176/appi.neuropsych.16090168 28238272

[88] Steinberg M Lyketsos CG. Atypical antipsychotic use in patients with dementia: managing safety concerns. Am J Psychiatry (2012) 169:900–6. 10.1176/appi.ajp.2012.12030342 22952071 PMC3516138

[89] Mendez MF Shapira JS McMurtray A Licht E. Preliminary findings: behavioral worsening on donepezil in patients with frontotemporal dementia. Am J Geriatr Psychiatry (2007) 15:84–7. 10.1097/01.JGP.0000231744.69631.33 17194818

[90] Chow TW. Treatment approaches to symptoms associated with frontotemporal degeneration. Curr Psychiatry Rep. (2005) 7:376–80. 10.1007/s11920-005-0040-5 16216158

[91] Vercelletto M Boutoleau-Bretonniere C Volteau C Puel M Auriacombe S Sarazin M. Memantine in behavioral variant frontotemporal dementia: negative results. J Alzheimers Dis. (2011) 23:749–59. 10.3233/JAD-2010-101632 21157021

[92] Boxer AL Knopman DS Kaufer DI Grossman M Onyike C Graf-Radford N. Memantine in patients with frontotemporal lobar degeneration: a multicentre, randomised, double-blind, placebo-controlled trial. Lancet Neurol. (2013) 12:149–56. 10.1016/S1474-4422(12)70320-4 23290598 PMC3756890

[93] Mocellin R Scholes A Walterfang M Looi JC Velakoulis D. Clinical update on frontotemporal dementia: diagnosis and treatment. Australas Psychiatry (2015) 23:481–7. 10.1177/1039856215582276 25907795

[94] Sanders DW Kaufman SK DeVos SL Sharma AM Mirbaha H Li A. Distinct tau prion strains propagate in cells and mice and define different tauopathies. Neuron (2014) 82:1271–88. 10.1016/j.neuron.2014.04.047 24857020 PMC4171396

[95] Yanamandra K Kfoury N Jiang H Mahan TE Ma S Maloney SE. Anti-tau antibodies that block tau aggregate seeding *in vitro* markedly decrease pathology and improve cognition *in vivo*. Neuron (2013) 80:402–14. 10.1016/j.neuron.2013.07.046 24075978 PMC3924573

[96] Wischik CM Harrington CR Storey JM. Tau-aggregation inhibitor therapy for Alzheimer's disease. Biochem Pharmacol. (2014) 88:529–39. 10.1016/j.bcp.2013.12.008 24361915

[97] Coughlin D Irwin DJ. Emerging diagnostic and therapeutic strategies for tauopathies. Curr Neurol Neurosci Rep. (2017) 17:72. 10.1007/s11910-017-0779-1 28785992 PMC5756477

[98] Mancini RS Wang Y Weaver DF. Phenylindanes in brewed coffee inhibit amyloid-beta and tau aggregation. Front Neurosci. (2018) 12:735. 10.3389/fnins.2018.00735 30369868 PMC6194148

[99] Buchholz K Schirmer RH Eubel JK Akoachere MB Dandekar T Becker K. Interactions of methylene blue with human disulfide reductases and their orthologues from Plasmodium falciparum. Antimicrob Agents Chemother. (2008) 52:183–91. 10.1128/AAC.00773-07 17967916 PMC2223905

[100] Heard DS Tuttle CSL Lautenschlager NT Maier AB. Repurposing proteostasis-modifying drugs to prevent or treat age-related dementia: a systematic review. Front Physiol. (2018) 9:1520. 10.3389/fphys.2018.01520 30425653 PMC6218672

[101] Wischik CM Edwards PC Lai RY Roth M Harrington CR. Selective inhibition of Alzheimer disease-like tau aggregation by phenothiazines. Proc Natl Acad Sci USA. (1996) 93:11213–8. 10.1073/pnas.93.20.11213 8855335 PMC38310

[102] Matsuoka Y Jouroukhin Y Gray AJ Ma L Hirata-Fukae C Li HF. A neuronal microtubule-interacting agent, NAPVSIPQ, reduces tau pathology and enhances cognitive function in a mouse model of Alzheimer's disease. J Pharmacol Exp Ther. (2008) 325:146–53. 10.1124/jpet.107.130526 18199809

[103] Morimoto BH Schmechel D Hirman J Blackwell A Keith J Gold M. A double-blind, placebo-controlled, ascending-dose, randomized study to evaluate the safety, tolerability and effects on cognition of AL-108 after 12 weeks of intranasal administration in subjects with mild cognitive impairment. Dement Geriatr Cogn Disord. (2013) 35:325–36. 10.1159/000348347 23594991

[104] Boxer AL Lang AE Grossman M Knopman DS Miller BL Schneider LS. Davunetide in patients with progressive supranuclear palsy: a randomised, double-blind, placebo-controlled phase 2/3 trial. Lancet Neurol. (2014) 13:676–85. 10.1016/S1474-4422(14)70088-2 24873720 PMC4129545

[105] Pedersen JT Sigurdsson EM. Tau immunotherapy for Alzheimer's disease. Trends Mol Med. (2015) 21:394–402. 10.1016/j.molmed.2015.03.003 25846560

[106] Medina M Avila J. New insights into the role of glycogen synthase kinase-3 in Alzheimer's disease. Expert Opin Ther Targets (2014) 18:69–77. 10.1517/14728222.2013.843670 24099155

[107] West T Hu Y Verghese PB Bateman RJ Braunstein JB Fogelman I. Preclinical and clinical development of ABBV-8E12, a humanized anti-tau antibody, for treatment of Alzheimer's disease and other tauopathies. J Prev Alzheimers Dis. (2017) 4:236–41. 10.14283/jpad.2017.36 29181488

[108] Panza F Solfrizzi V Seripa D Imbimbo BP Lozupone M Santamato A. Tau-centric targets and drugs in clinical development for the treatment of Alzheimer's disease. Biomed Res Int. (2016) 2016:3245935. 10.1155/2016/3245935 27429978 PMC4939203

[109] O'Brien JT Holmes C Jones M Jones R Livingston G McKeith I. Clinical practice with anti-dementia drugs: a revised (third) consensus statement from the British Association for Psychopharmacology. J Psychopharmacol. (2017) 31:147–68. 10.1177/0269881116680924 28103749

[110] Lagier-Tourenne C Baughn M Rigo F Sun S Liu P Li HR. Targeted degradation of sense and antisense C9orf72 RNA foci as therapy for ALS and frontotemporal degeneration. Proc Natl Acad Sci USA. (2013) 110:E4530–9. 10.1073/pnas.1318835110 24170860 PMC3839752

[111] Kramer NJ Carlomagno Y Zhang YJ Almeida S Cook CN Gendron TF. Spt4 selectively regulates the expression of C9orf72 sense and antisense mutant transcripts. Science (2016) 353:708–12. 10.1126/science.aaf7791 27516603 PMC5823025

[112] DeVos SL Goncharoff DK Chen G Kebodeaux CS Yamada K Stewart FR. Antisense reduction of tau in adult mice protects against seizures. J Neurosci. (2013) 33:12887–97. 10.1523/JNEUROSCI.2107-13.2013 23904623 PMC3728694

[113] Cenik B Sephton CF Dewey CM Xian X Wei S Yu K. Suberoylanilide hydroxamic acid (vorinostat) up-regulates progranulin transcription: rational therapeutic approach to frontotemporal dementia. J Biol Chem. (2011) 286:16101–8. 10.1074/jbc.M110.193433 21454553 PMC3091219

[114] Capell A Liebscher S Fellerer K Brouwers N Willem M Lammich S. Rescue of progranulin deficiency associated with frontotemporal lobar degeneration by alkalizing reagents and inhibition of vacuolar ATPase. J Neurosci. (2011) 31:1885–94. 10.1523/JNEUROSCI.5757-10.2011 21289198 PMC6623716

[115] Whitwell JL Weigand SD Boeve BF Senjem ML Gunter JL DeJesus-Hernandez M. Neuroimaging signatures of frontotemporal dementia genetics: C9ORF72, tau, progranulin and sporadics. Brain (2012) 135(Pt 3):794–806. 10.1093/brain/aws001 22366795 PMC3286334

[116] Alberici A Archetti S Pilotto A Premi E Cosseddu M Bianchetti A. Results from a pilot study on amiodarone administration in monogenic frontotemporal dementia with granulin mutation. Neurol Sci. (2014) 35:1215–9. 10.1007/s10072-014-1683-y 24569924

